# Bone mineral density in elite rowers

**DOI:** 10.1186/2052-1847-7-S1-O6

**Published:** 2015-08-11

**Authors:** Bronwen Lundy, Larissa Trease, Drew K Michael

**Affiliations:** 1 Australian Institute of Sport, Canberra, Australia; 2 Principal Medical Officer, Rowing Australia, Canberra, Australia; 3 Department of Physiotherapy, Faculty of Health, University of Canberra, Australia; 4 Australian Centre for Research into Injury in Sport and its Prevention (ACRISP), Federation University, Australia

## Background

Bone mineral density (BMD) is known to be dependent on the loading pattern associated with a particular sport. High impact sports increases BMD at loaded sites with low impact sports having largely neutral findings [1]. The influence of high level rowing training has not been well explored with relatively small samples within a single category or discipline [2–5].

## Methods

Subjects (n=125) were internationally competitive. Between 2011-2014 BMD was taken at the lumbar spine (L1-L4) and left femur, by Dual-energy X-ray absorptiometry (DXA, Lunar Prodigy, GE Healthcare), using the same scanner, and a qualified technician. Ethics was approved by the Australian Institute of Sport Human Ethics committee. Subjects gave prior written informed consent. Descriptive statistics are reported as mean ± standard deviation (range), Z-score and T-score. Statistical analysis was performed using independent samples t-test, significance set at p<0.05.

## Results

A summary of findings is shown in Table 1. Overall, 5.6 % of rowers had Z ≤-1 at the spine and 1.6% at the femur with none Z <-2. Both spine and femur BMD, T and Z scores were lower for female lightweights than heavyweights. Male spine BMD and T score and femur T score was lower for lightweights relative to heavyweights.

**Table 1:** BMD in males and female rowers by weight category. Data are expressed mean ± standard deviation (range).

	Males			Females		
	Overall	Lightweight	Heavyweight	Overall	Lightweight	Heavyweight
n	72	31	41	53	20	33
Spine						
g/cm^2^	1.33 ± 0.13	1.27 ± 0.10 *	1.38 ± 0.12	1.29 ± 0.14	1.19 ± 0.09 *	1.35 ± 0.14
	(1.07 -1.67)	(1.07 -1.46)	(1.09 – 1.67)	(1.05-1.67)	(1.05 - 1.33)	(1.07 - 1.67)
T score	0.9 ± 1.1	0.2 ± 0.9 *	1.3 ± 1.0	0.6 ± 1.1	-0.3 ± 0.7 *	1.0 ± 1.1
	(-1.3-3.5)	(-1.3 - 1.7)	(-1.1 – 3.5)	(-1.2-3.5)	(-1.2 - 0.9)	(-1.0 - 3.5)
Z score	0.7 ±1.0	0.5 ± 1.9	0.8 ± 1.0	0.4 ±1.0	0.1 ± 0.7 *	0.7 ± 1.0
	(-1.5-3.2)	(-1.2 – 1.9)	(-1.5 – 3.2)	(-1.3-3.3)	(-1.0 – 1.2)	(-1.3-3.3)
Femur						
g/cm^2^	1.19 ± 0.13	1.16 ± 0.13	1.21 ± 0.12	1.12 ±0.13	1.05 ± 0.09 *	1.17 ± 0.13
	(0.97-1.58)	(1.00 -1.54)	(1.02 - 1.58)	(0.87-1.61)	(0.87 -1.23)	(0.96 - 1.61)
T score	0.7 ± 1.0	0.4 ± 0.9	1.0 ± 0.9	0.5 ±0.9	0.0 ± 0.7 *	0.8 ± 0.8
	(-1 – 3.8)	(-1.0 – 2.2)*	(-0.6 - 3.8)	(-1.4-3.2)	(-1.4 – 1.3)	(-0.7 - 3.2)
Z score	0.5 ± 0.9	0.4 ± 0.9	0.5 ± 1.0	0.3 ±0.78	0.2 ± 0.8 *	0.4 ± 0.8
	(-1 – 3.4)	(-0.9 – 2.3)	(-1.0 - 3.4)	(-1.1-3.0)	(-1.1 - 1.6)	(-0.8 - 3.0)

* significantly lower than for heavyweights within the same gender (p<0.05)

## Conclusion

BMD of elite rowers appears to fall largely within the optimal range for the general population however lightweight rowers, tended to have lower BMD than their heavyweight counterparts at all measured sites at the spine and for females also at the femur.
